# Supplementary material for: Epidemiology and reporting characteristics of preclinical systematic reviews
Source: PLoS Biol. 2021 May 5;19(5):e3001177. doi: 10.1371/journal.pbio.3001177 (PMC8128274; doi:10.1371/journal.pbio.3001177)
Supplement: S1 Checklist — (DOCX) [file pbio.3001177.s001.docx]

**S1 Checklist.** Reporting checklist to assess the state of reporting within preclinical systematic reviews.

| Section | # | Item |
| --- | --- | --- |
| Title | 1 | Identify the report as systematic review in title |
|  | 2 | Identify that the report contains animal data in title (preclinical, *in vivo* or synonym) |
| Intro | 3 | Describe the human condition being modelled (e.g. describe what is already known) |
|  | 4 | Describe the biological rationale for testing the intervention (e.g. how would the intervention affect the condition) |
|  | 5 | Provide an explicit statement of the question(s) the review addresses (specify the main objectives of the review, ideally in PICO format) |
| Methods | 6 | Indicate whether a review protocol was registered *a priori* |
|  | a | Where can the protocol be accessed and indicate the name of the protocol registry OR state that it is not available |
|  | b | Indicate any deviations from the protocol OR that there were no deviations |
|  | 7 | Eligibility criteria: Describe the animal species to be included in the review (e.g. only mice, vertebrates, large animals) |
|  | 8 | Eligibility criteria: Describe the animal model to be included in the review (methods of disease induction, age, sex, etc.) |
|  | 9 | Eligibility criteria: Describe the intervention/exposure of interest |
|  | 10 | Eligibility criteria: Describe the comparators and/or control population |
|  | 11 | Eligibility criteria: Describe the primary outcomes of interest (what is being measured/assessed in primary studies) |
|  | 12 | Eligibility criteria: Describe the timing (prevention vs rescue) of intervention, IF applicable |
|  | 13 | Indicate where a full search strategy of all data bases OR representative search strategy can be accessed |
|  | 14 | Describe inclusion limits (years conducted, language, AND publication type) |
|  | 15 | Describe the study screening/selection process |
|  | a | Report the platform used to screen and select studies (Excel, Access, DistillerSR, SyRF) |
|  | 16 | State the number of independent screeners |
|  | 17 | Describe methods for extracting numerical data from reports (e.g. data in bar graph, or non-text presentation), IF applicable * |
|  | a | Report the platform and tools used to extract numerical data (Graph2data, Engauge) |
|  | 18 | Report number of independent reviewers extracting data |
|  | 19 | Describe methods and tool used to measure study quality/risk of bias in individual studies (e.g. SYRCLE tool, CAMARADES tool) |
|  | 20 | Describe methods to assess construct validity in individual studies |
|  | 21 | Describe methods for assessing publication bias of included studies, IF applicable |
|  | 22 | Describe methods for synthesizing the quantitative effect measures of included studies (e.g. risk ratio, mean difference), IF applicable * |
|  | 23 | Describe methods for any data transformation needed to make extracted data suitable for analysis (e.g. only sample size range), IF applicable * |
|  | 24 | Describe methods for handling shared control groups (common issue in analysis of preclinical studies), IF applicable * |
|  | 25 | Describe methods for assessing heterogeneity between individual studies, IF applicable * |
|  | 26 | Describe methods for handling effect sizes over multiple time points (e.g. used all time points or latest time point), IF applicable * |
|  | 27 | Describe methods for sub-group and sensitivity analysis, IF applicable * |
| Results | 28 | Report the number of included reports (individual references/publication) included in the review |
|  | a | Provides a list or table of individual studies with data or references |
|  | 29 | Report the number of eligible experiments included in the analysis (eligible animal experiments in individual reports) |
|  | 30 | Include a PRISMA flow diagram (or equivalent) of study selection process |
|  | 31 | Study characteristics: Report animal species |
|  | 32 | Study characteristics: Report animal model details (e.g. method of disease induction, age, sex) |
|  | 33 | Study characteristics: Report a measure of the sample size (e.g. total number or mean number of animals) |
|  | 34 | Study characteristics: Report intervention/exposure details (timing, dose) |
|  | 35 | Study characteristics: Report study design/intention (pharmakinetic, mechanistic, efficacy) |
|  | 36 | Report the risk of bias of the primary studies (individual studies/across outcomes) |
|  | 37 | Report the outcome effects of primary studies (forest plot if applicable), IF applicable * |
|  | 38 | Report the confidence intervals of outcomes for the included studies, IF applicable * |
|  | 39 | Report any measure of heterogeneity between studies, IF applicable * |
|  | 40 | Report the results of sub-group and sensitivity analysis, IF applicable * |
|  | 41 | Report the results of publication bias, OR report that it was not possible/done |
| Discussion | 42 | Discuss the impact of the risk of bias of the primary studies |
|  | 43 | Discuss the limitations (i.e. limitation of primary studies and/or outcomes included) |
|  | 44 | Discuss the limitations of the systematic review |
| Other | 45 | Include the funding source(s) of the systematic review |
|  | 46 | Report any data sharing, OR that there was no data sharing |
| * Reporting item is not applicable to systematic reviews that did not perform a quantitative synthesis. For reviews that did not perform a quantitative synthesis, these items receive an NA. | | |
