# Supplementary material for: Epidemiology and reporting characteristics of preclinical systematic reviews
Source: PLoS Biol. 2021 May 5;19(5):e3001177. doi: 10.1371/journal.pbio.3001177 (PMC8128274; doi:10.1371/journal.pbio.3001177)
Supplement: S1 Appendix — (DOCX) [file pbio.3001177.s002.docx]

**S1 Appendix.** Systematic search strategy.

Database: Embase Classic+Embase <1947 to 2019 March 20>, Ovid MEDLINE(R) ALL <1946 to March 20, 2019>

Search Strategy:

--------------------------------------------------------------------------------

1     meta-analysis.pt. (98487)

2     (meta-analy* or metaanaly* or metanaly* or met analy*).ti,ab. (333373)

3     "meta-analysis as topic"/ (42912)

4     or/1-3 (378559)

5     review.pt,ab. (6228648)

6     systematic.tw. (694670)

7     5 and 6 (349992)

8     (systematic* adj5 (review* or overview*)).ti,ab. (342285)

9     4 or 7 or 8 (636452)

10     (comment or editorial or letter).pt. (3351144)

11     9 not 10 (617380)

12     limit 11 to animals (17717)

13     exp Animal Experimentation/ (2357279)

14     exp models, animal/ (1764737)

15     experiment*.ti,ab. (4181761)

16     limit 15 to animals (1617517)

17     13 or 14 or 16 (4251842)

18     11 and 17 (7489)

19     12 or 18 (20277)

20     meta-analysis.pt. (98487)

21     (meta-analy* or metaanaly* or metanaly* or met analy*).tw,kw. (337365)

22     "meta-analysis as topic"/ (42912)

23     or/20-22 (382185)

24     review.pt,ab. (6228648)

25     systematic.tw,kw. (697610)

26     24 and 25 (351511)

27     (systematic* adj5 (review* or overview*)).ti,ab. (342285)

28     23 or 26 or 27 (639738)

29     (comment or editorial or letter).pt. (3351144)

30     28 not 29 (620334)

31     limit 30 to animals (17844)

32     exp Animal Experimentation/ (2357279)

33     exp models, animal/ (1764737)

34     experiment*.tw,kw. (4303640)

35     limit 34 to animals (1672077)

36     32 or 33 or 35 (4300654)

37     30 and 36 (7555)

38     31 or 37 (20419)

39     limit 38 to yr="2018" (1895)

40     (2018* not (2018010* or 2018011* or "20180120" or "20180121")).dt. (1206782)

41     38 and 40 (749)

42     39 or 41 (1961)

43     42 use medall (1085)

44     exp "animal experiment"/ or (animal* and stud*).tw. or exp "animal model"/ or (animal* and model*).tw. (4142400)

45     environmental exposure.tw. (14612)

46     exp environmental exposure/ (380665)

47     exp toxicology/ (100105)

48     toxicology.tw. (62210)

49     exp genetic toxicology/ (970)

50     exp comparative toxicology/ (260)

51     mutagen*.tw. (235710)

52     exp mutagenic agent/ (18732)

53     carcinogen.tw. (50195)

54     exp carcinogen/ (145604)

55     drug toxicity.fs. (537731)

56     adverse drug reaction.fs. (1260505)

57     side effect.fs. (848786)

58     or/45-57 (2586499)

59     44 and 58 (299226)

60     (meta and analy*).tw. (339980)

61     meta analysis/ (256415)

62     (systematic* and review*).tw. (403251)

63     "systematic review"/ (299056)

64     or/60-63 (697228)

65     59 and 64 (2030)

66     (2018* not (2018010* or 2018011* or "20180120" or "20180121")).dc. (1613924)

67     65 and 66 (135)

68     67 use emczd (135)

69     43 or 68 (1220)

70     remove duplicates from 69 (1190)

71     70 use medall (1085)

72     70 use emczd (105)

Databases: TOXLINE

Query: ( meta analysis AND animal ) AND 1900:2017 [yr] AND 201613:201801 [em] AND NOT PubMed [org] AND NOT pubdart [org]

Singular and plural forms were searched. = 83

Databases: TOXLINE

Query: ( systematic review AND animal ) AND 201613:201801 [em] AND NOT PubMed [org] AND NOT pubdart [org]

Singular and plural forms were searched. = 27
