# Supplementary material for: Epidemiology and reporting characteristics of preclinical systematic reviews
Source: PLoS Biol. 2021 May 5;19(5):e3001177. doi: 10.1371/journal.pbio.3001177 (PMC8128274; doi:10.1371/journal.pbio.3001177)
Supplement: S1 Table — (DOCX) [file pbio.3001177.s003.docx]

**S1 Table.** Countries of the corresponding authors that have published a preclinical systematic review.

| Country of corresponding author | Number (%), of *n* = 442 |
| --- | --- |
| United States | 59 (13) |
| Brazil | 52 (12) |
| China | 49 (11) |
| United Kingdom | 44 (10) |
| Netherlands | 43 (10) |
| Iran | 28 (6) |
| Canada | 27 (6) |
| Denmark | 22 (5) |
| Germany | 16 (4) |
| Australia | 13 (3) |
| France | 10 (2) |
| Belgium | 9 (3) |
| Italy | 9 (2) |
| Spain | 7 (2) |
| Switzerland | 7 (2) |
| Greece | 4 (0.9) |
| Japan | 4 (0.9) |
| Portugal | 4 (0.9) |
| Egypt | 3 (0.7) |
| Malaysia | 3 (0.7) |
| India | 2 (0.5) |
| Ireland | 2 (0.5) |
| Israel | 2 (0.5) |
| Mexico | 2 (0.5) |
| Norway | 2 (0.5) |
| Sweden | 2 (0.5) |
| Albania | 1 (0.2) |
| Argentina | 1 (0.2) |
| Austria | 1 (0.2) |
| Bulgaria | 1 (0.2) |
| Chile | 1 (0.2) |
| Finland | 1 (0.2) |
| Iceland | 1 (0.2) |
| Korea | 1 (0.2) |
| Poland | 1 (0.2) |
| Romania | 1 (0.2) |
| Saudi Arabia | 1 (0.2) |
| Slovak Republic | 1 (0.2) |
| Swaziland | 1 (0.2) |
| Thailand | 1 (0.2) |
| United Arab Emirates | 1 (0.2) |
| Vietnam | 1 (0.2) |
