# Supplementary material for: Epidemiology and reporting characteristics of preclinical systematic reviews
Source: PLoS Biol. 2021 May 5;19(5):e3001177. doi: 10.1371/journal.pbio.3001177 (PMC8128274; doi:10.1371/journal.pbio.3001177)
Supplement: S2 Table — (DOCX) [file pbio.3001177.s004.docx]

**S2 Table.** Animal species reported within the primary studies of the preclinical systematic reviews.

| Category | Characteristic | Number (%), of *n* = 442 |
| --- | --- | --- |
| Animal species | Rat | 346 (78) |
|  | Mouse | 287 (65) |
|  | Rabbit | 127 (29) |
|  | Dog | 84 (19) |
|  | Swine | 77 (17) |
|  | Sheep | 52 (12) |
|  | Non-human primate | 44 (10) |
|  | Goat | 20 (4) |
|  | Cat | 18 (4) |
|  | Hamster | 16 (4) |
|  | Guinea pig | 15 (3) |
|  | Mini swine | 14 (3) |
|  | Avian | 11 (3) |
|  | Cattle | 10 (2) |
|  | Horse | 10 (2) |
|  | Fish | 9 (2) |
|  | Gerbil | 3 (0.7) |
|  | Reptile | 3 (0.7) |
|  | Not reported | 23 (5) |
| Number of species | One | 43 |
|  | Two | 85 |
|  | Three | 55 |
|  | Four | 35 |
|  | Five | 13 |
|  | Six | 16 |
|  | Seven | 10 |
|  | Eight | 4 |
|  | Nine | 2 |
