# Supplementary material for: Epidemiology and reporting characteristics of preclinical systematic reviews
Source: PLoS Biol. 2021 May 5;19(5):e3001177. doi: 10.1371/journal.pbio.3001177 (PMC8128274; doi:10.1371/journal.pbio.3001177)
Supplement: S4 Table — (DOCX) [file pbio.3001177.s006.docx]

**S4 Table.** Disease domains investigated in the preclinical systematic reviews in sub-group of studies performing quantitative analyses.

| Category | Characteristic | Number (%), of *n* = 44 |
| --- | --- | --- |
| Type of disease domain | Musculoskeletal system and connective tissue | 7 (16) |
|  | Nervous system | 7 (16) |
|  | Cardiovascular system | 9 (20) |
|  | Endocrine, nutritional and metabolic diseases | 3 (7) |
|  | Cancer | 0 (0) |
|  | Toxicology | 3 (7) |
|  | Mental and behaviour | 5 (11) |
|  | Genitourinary system | 3 (7) |
|  | Skin and subcutaneous tissue | 2 (5) |
|  | Digestive system | 1 (2) |
|  | Critical illness | 4 (9) |
|  | Infectious and parasitic diseases | 1 (2) |
|  | Respiratory system | 2 (5) |
|  | Pain and analgesia | 0 (0) |
|  | General and whole-body health | 1 (2) |
|  | Conditions originating in the perinatal period | 1 (2) |
|  | Pharmacokinetic, biological activity and dose-response | 0 (0) |
|  | Blood and immune disorders | 2 (5) |
|  | Eye | 1 (2) |
|  | Mouth | 1 (2) |
|  | Congenital malformations | 0 (0) |
|  | Surgery and imaging techniques | 0 (0) |
|  | Auditory system | 0 (0) |
| Number of disease domains per review | One | 36 (82) |
|  | Two | 8 (18) |
|  | Three | 0 (0) |
|  | >Three | 0 (0) |
