# Supplementary material for: Epidemiology and reporting characteristics of preclinical systematic reviews
Source: PLoS Biol. 2021 May 5;19(5):e3001177. doi: 10.1371/journal.pbio.3001177 (PMC8128274; doi:10.1371/journal.pbio.3001177)
Supplement: S5 Table — (DOCX) [file pbio.3001177.s007.docx]

**S5 Table.** Intervention and intervention sub-groups evaluated in the preclinical systematic reviews in sub-group of studies performing quantitative analyses.

| Intervention  Number (%), of *n* = 44 | Sub-group | Number (%), of *n* = 44 |
| --- | --- | --- |
| Pharmacological  25 (60) | NA | |
| Non-pharmacological  19 (40) | Cell therapy | 9 (20) |
|  | Surgery or invasive procedures | 1 (2) |
|  | Medical physics | 2 (5) |
|  | Dietary interventions | 2 (5) |
|  | Blood transfusions or modifications | 0 (0) |
|  | Animal model validation | 0 (0) |
|  | Tactile stimulus interventions | 0 (0) |
|  | Exercise and physical activity | 0 (0) |
|  | Oxygen therapy | 1 (2) |
|  | Gene therapy | 1 (2) |
|  | Other | 3 (70) |
