# Supplementary material for: Epidemiology and reporting characteristics of preclinical systematic reviews
Source: PLoS Biol. 2021 May 5;19(5):e3001177. doi: 10.1371/journal.pbio.3001177 (PMC8128274; doi:10.1371/journal.pbio.3001177)
Supplement: Attachment — Submitted filename: PLOS Biology Response to Reviewers.docx [file pbio.3001177.s008.docx]

**Dear Dr. Roberts,**

**We sincerely thank you and the reviewers for considering our work and your overall positive reception. We have addressed all comments, extracted additional data and performed further analyses requested. These have resulted in significant revisions. We hope you will find the manuscript has been strengthened as a result.**

**As you may be aware, the updated PRISMA 2020 reporting guidelines will be published shortly (several members of our group are authors). Thus, we believe the results of this study will be highly topical and well-received by the larger meta-research community.**

**Sincerely,**

**Victoria Hunniford and Manoj Lalu on behalf of all co-authors**

***Response to Handling Editor; Dr. Roland Roberts***

Comment: …You'll see that the reviewers are broadly positive about your study, but between them they raise a number of concerns about possible coding artefacts, the need to assess trends over time, more discussion of remedies, clarifications of the motivation and some choices made, and the need for an update to the data (we note that the search date is nearly two years ago). These issues will need to be addressed for further consideration.

**Response:**

***Coding artifacts:***

**We have carefully reviewed and addressed any discrepancies in the data between the results, figures, and discussion that were raised by Reviewer #1 (Dr. Rooney). Additionally, we have elaborated on our methods for conducting the reporting assessment. We believe the additional information provided demonstrates that this process was robust and resulted in a very high interrater correlation.**

***Trends over time:***

**We have now added a formal comparison (z-test) of our sample to the previous assessment of reporting by Mueller et al 2014.^1^ These statistics are now included in the Discussion:**

**“Mueller et al [5] found that 62% of the reviews they identified provided a flow diagram of included studies; we found that 86% did so (Z-test, p<0.0001). Similarly, reporting of funding also improved (78% vs 38%; Z-test, p<0.00001). Fourteen percent of reviews assessed publication bias in the previous reporting assessment, while 25% of reviews reported the results of publication bias in our updated evaluation (Z-test, p=0.01).”**

***Discussion of remedies:***

**We agree with Dr. Roberts that this is critical elaborate upon. We have added some commentary around this in the Discussion:**

**“In addition to the development of reporting guidelines, other initiatives must be considered to improve the state of reporting and quality of preclinical systematics reviews. Journals, funders, and reviewers could contribute to this improvement by advocating and appealing for data sharing, registration of protocols a priori, encouraging the use of tools and resources created by SYRCLE, CAMARADES, and similar groups. Moreover, reporting transparency could be improved by moving beyond simple endorsement and instead enforcing the use of a reporting checklist.”**

***Clarification of motivation:***

**The motivation of this work is to inform a larger research program aiming to develop specific guidelines for the reporting of *in vivo* systematic reviews of interventions. This process of performing a cross-sectional study prior to guideline developments mirrors methods used by Moher *et al^2^* when creating the original PRISMA guidelines. This also follows best practices for reporting guideline development^3^.**

**We have first clarified distinctions between clinical and preclinical systematic reviews in our introduction:**

**“There are fundamental differences between preclinical and clinical systematic reviews of interventions. For instance, subjects are laboratory animals instead of humans, and studies employ different procedures compared with clinical studies (e.g. inducing the disease in animals to mimic the human condition, sacrificing animals to evaluate outcomes, etc.). Therefore, species and strain specific effects, along with unique elements of data abstraction may need to be considered. In addition, preclinical systematic reviews are sometimes performed as precursors to attempted clinical translation of a novel therapy. Therefore, in addition to risk of bias, exploring construct validity of included primary studies (i.e. how well the animal models mimic the disease of interest) may provide important insights on the potential for translation.[9] Finally, it is commonplace to include primary studies with multiple experiments, each with many experimental arms; this requires both significant efforts to identify specific data to include, as well as special considerations for how data will be handled for analysis.”**

**In addition, we have also clarified in last paragraph of the Introduction that this assessment is separate from the future development of a PRISMA extension for preclinical systematic reviews:**

**“… This assessment will help inform the future development of reporting guidelines designed specifically for preclinical systematic reviews, as an extension to the PRISMA statement.”**

***Need for an update:***

**We would respectfully suggest that the update mentioned by Reviewer #2 (Dr. Langendam) may not be warranted since there have been no major initiatives to address preclinical SR reporting since the search was conducted. Thus, our co-author group believes there is no reason to think that updated results from 2019-2020 would be meaningfully different. Moreover, an update would not be feasible to complete in a short period of time (please note that this project is being conducted by our team without funding) and it is important to complete this particular study as the planned Delphi exercise to develop an extension to PRISMA will require our available resources. Nonetheless, we have now acknowledged this limitation in our Discussion:**

**“A potential limitation is the timeframe of our sample, as we included reviews from 2015 to 2018 inclusively. Though we chose this sample to capture the state of reporting in preclinical systematic reviews after the previous assessment in 2014, we acknowledge that the state of reporting may have changed from 2018; however, it is important to note that no major initiatives to address systematic review reporting have occurred since that time.”**

***Response to peer-reviewers***

**Reviewer #1: [identifies himself as Kieron Rooney]**

Specific comments requiring response:

Lines 266-267 present a result in a different manner / context in which the checklist was implemented and this raises a concern regarding the process in which multiple authors were stated as implementing the assessment with consensus or third review. For example, we are given a result here of "eligible intervention timing (prevention vs rescue)" Yet in the checklist the qualifier "prevention vs rescue" is in regard to "intended goal of the intervention". I am concerned then that assessors of the articles may have scored this item differently as a result of inconsistent procedure. Can the authors please confirm if item 12 was assessed consistently between authors AND was the "prevention vs rescue" element assessed regarding whether it appeared within the context of intervention timing or intended goal of the intervention. Finally, can the authors also confirm how the potential for such a discrepancy was confirmed prior to submission.

**Response: For all reporting assessment items, we conducted pilot sessions with reviewers. All reviewers were provided one-on-one coaching through questions by a senior research group member. Indeed, our high inter-reviewer agreement reflects the rigour of this process (kappa = 0.89). We added the following to the methods: “The reporting assessment was piloted on 7 reviews from the sample to ensure reviewers were evaluating the reporting items consistently.”**

**For the particular question identified by Dr. Rooney, the ‘intended goal’ was in fact a description of timing as the only two answers that extractors could choose were either ‘prevention’ and/or ‘rescue’ (i.e. this was a ‘checkbox’ question).**

**The discrepancy in wording from the results versus the checklist reflects co-author feedback on a draft of the manuscript; it was thought that simplifying the wording might add clarity.**

**We see this has caused confusion – thus we have edited the checklist wording (Supplemental Material S4) to reflect the true intention of the question. Again, given that the only two answers the extractors could select were related to timing, we hope Dr. Rooney agrees this edit will add clarity and assist others in using our list for future evaluations of reporting.**

**Comment:** Checklist item 6 states to assess only whether or not a protocol was registered a priori however the results on lines 261-262 identify that it was also identified if a database was indicated. In this instance then item 6 should be updated as identifying 1) was a protocol registered and 2) is the database or ID provided so as to better represent how it must have been implemented by the authors here.

**Response: The full checklist can be found in Supplemental Material S4. Checklist item 6 “Indicate whether a review protocol was registered *a priori*” has a nested question, the first being 6a “Where can the protocol be accessed and indicate the name of the protocol registry OR state that it is not available”.**

**Comment:** Checklist item 20 - construct validity is identified as being described 0% in all studies. However, I wonder if this is simply an artefact of the checklist placing this item in the methods section. As an author of pre-clinical SRs reporting on interventions related to human clinical conditions, this is an item my colleagues and I have referred to in the discussion in the absence of a strong tool for assessing construct validity. The authors of this manuscript identify in the discussion that this concept is an emerging area of assessment. I wonder if the result presented here was re-assessed with a critical eye over attempts by authors of the SR to comment on construct validity in the discussion would identify a different outcome to be reported in this manuscript? Could the authors comment on why that item was placed only in the methods, and whether or not - even if the SRs reviewed included comment on construct validity in discussion - they scored 0 and whether or not reviewing the discussion sections of the included articles now and reporting on item 20 as an alternate discussion item could be a worthwhile adventure.

**Response: We agree that authors of the reviews may have speculated on construct validity in the Discussion. We felt it was important to place this item in the methods, however, as we were specifically interested in identifying if the authors had evaluated this in an objective and potentially reproducible manner. Dr. Rooney raises an interesting point that our co-author group felt was worth speculating on further. This, along with further thoughts on construct validity, are now addressed in the Discussion:**

**“In addition, items that are unique to preclinical systematic reviews (e.g. construct validity) were not evaluated objectively. Although authors may speculate on translational potential in the discussion section of papers, it remains rare for reproducible frameworks to be used to evaluate it. In part, this may reflect a lack of consensus on the definition of ‘construct validity’; although the concept of translation is often discussed, variable terms and approaches have been suggested (e.g. translation as a component of external validity;^4^ using a unique term such as ‘translational validity’,^5^ etc.). Defining this concept further through future research may be warranted.”**

**Comment:** Lines 298-312 and figure 5E identify that the checklist items presented as supplementary 4 are a conflated list of two checklist - there are some questions apparently only relevant to SRs with quantitative analysis. This should have been more explicitly articulated in the method section and then also in the checklist items that are only relevant to some SRs but not all should be clearly identified. If this work is (as the authors seem to hope) to inform a PRISMA extension, then it is vital that any potentially "irrelevant" questions are clearly articulated.

**Response: This has been more clearly reported in the methods, checklist, results, and figures:**

**Methods:**

**“…Items were assessed in each review as being reported (‘yes’) or not (‘no’), or not applicable to the review (‘NA’). If a review did not contain quantitative data (i.e. no meta-analysis), ‘NA’ was selected for all items relating to quantitative data/analysis (e.g. report methods for extracting numerical data from report)...”**

**Checklist (Supplemental Material S4):**

 “***Reporting item is not applicable to systematic reviews that did not perform a quantitative synthesis. For reviews that did not perform a quantitative synthesis, these items receive an NA.”**

**Results:**

**“For the reviews that did not perform a quantitative analysis, the quantitative items were evaluated as ‘NA’ (as described in the methods).”**

**Figures: Legends now provided for figure 5A-E.**

**Comment:** Lines 298-312 sub-group analysis: I would like to at least know how many of the 42 reviews now included in a sub-analysis implemented a pharmacological or non-pharmacological intervention. The potential need for a re-analysis / updated presentation of the results in this section then may be needed or at least commented on if the authors suspect their results here may have been impacted by the distribution across those interventions. Further, an identification of the disease domains for the 110 and 42 should be provided so one can tell if the results presented may have been impacted by a non-representative sub-group relative to the original 442.

**Response: We agree with Dr. Rooney that this represents an important piece of information. Two supplementary tables have been created with this information (mirroring tables 2 and 3 from the main document) to display this information. This information is also summarized briefly in the Results:**

**“The 44 quantitative reviews investigated 17 of the 23 diseases domains: cardiovascular system disorders (9 reviews; 20%), followed by musculoskeletal and connective tissue disorders, and nervous system disorders (7; 16% each). Twenty-five (60%) quantitative reviews investigated pharmacological interventions, and 19 (40%) investigated non-pharmacological interventions. Characteristics of the 44 quantitative reviews are found in supporting information S5 Table and S6 Table.”**

**Thus, the top three disease domains in the larger sample are also the top three in our sub-analysis. In addition, the proportion of pharmacological/non-pharmacological interventions remains similar (60/40% in subanalysis, versus 54/46% in larger sample).**

**Comment:** Checklist item ?? - I do not have a number to cross reference to as this item does not yet exist, but I'd have appreciated a specific question that asks whether or not the quantitative analysis in SRs grouped species or performed sub-group analyses as the contribution of species differences can often be ignored in analyses of heterogeneity.

**Response: We agree that this is an important question to explore, however it was one that was not included in our evaluation. The creation of this reporting assessment checklist was truly a group effort, with all co-authors providing input on items and agreeing on items to include/exclude.**

**It is important to distinguish this reporting assessment checklist from the eventual PRISMA extension we will be developing. We are currently in the process of creating a list of items to use for the Delphi survey of experts. Given this feedback from Dr. Rooney, we will incorporate his suggestion on subgroups in a list to be discussed and voted on.**

**Comment:** I have a concern regarding how the determination of the purpose of the SRs included was performed. It is not clear, if the authors of the original SR had to explicitly state they performed the SR for the purpose of informing construct validity / pre-clinical scope / translation to humans OR if it was the authors of this manuscript that inferred that purpose on the SR. For example, many pre-clinical SRs of interventions that would have met this inclusion criteria have been conducted NOT for the explicit purpose of informing human clinical practice, but rather to synthesise the current existing evidence to inform a future study design - best model, most appropriate species, etc. I would like to know if the authors of this manuscript specifically assessed if the originating SR authors explicitly stated an intent that the SR would enhance or inform translation to humans, or if that was inferred by the current authors. If it was simply assumed by the current authors then this should be included as a limitation of the analysis since it is possible that within the 75% of SRs not assessed there are better conducted SRs as their purpose was different.

**Response: By selecting animal studies of therapeutic interventions, we felt the vast majority of studies would be used to inform downstream translation to humans. Nonetheless, we recognize this limitation to our approach, and it is acknowledged in the Discussion.**

**“In addition, some of the items in our reporting assessment may** **not be generalizable to other forms of preclinical systematic reviews (e.g. those not focused on therapeutic interventions).”**

Typographical:

Line 120: delete "was defined" which is repetitive

**Response: Corrected.**

Lines 199 - 205 and figure 1. There are distinct differences in the numbers reported in the text and figure that need to be checked and corrected. For example - line 199 states 1549 papers were excluded, however figure 1 identifies 1585; line 200 states 807 papers were retrieved but the figure says 771; line 201 stated 325 papers were excluded but figure 1 states 329. Line 209 states 14% table 1 says 15% Line 229 states 21 different disease domains; Table 2 identifies 23

**Response: Corrected.**

Editorial:

Lines 146 - 148 and elsewhere for example line 168. When describing how the articles were screened and excluded etc it would be good to specifically identify which authors participated in the various tasks rather than just say "two authors …" or "we".

**Response: Given the number of authors that participated in each task we thought it would decrease readability of the methods. Instead, we specify contributions by using CRediT taxonomy on the title page.**

Line 236 and 237: the use of the word "had" seems so lax and "reported" would be more accurate representation.

**Response: Corrected.**

Lines 238 - 241 represent the relative % of occurrence to the 203 non-pharmacological sub-set yet table 3 is relative to the total 442 excluded. I don't really care which is used, but only one reference point should be used for consistency.

**Response: Corrected.**

Figure 4: I think the relative number of the final 110 that are pharmacological and non-pharmacological should be identified.

**Response: Excellent point. We added the additional information in the text and figure 4:**

**“To assess the completeness of reporting within preclinical systematic reviews, we selected a random sample of 110 articles (25% of the 442 identified reviews); 64 of which evaluated pharmacological interventions and 46 evaluated non-pharmacological interventions (Fig. 4).”**

Line 337: the sentence that ends at "increase" on line 376 is broken by the inclusion of "compared to a previous review published in 2014". I think this first half of line 337 could be deleted.

**Response: Corrected.**

**Reviewer #2: [identifies herself as Miranda W. Langendam]**

**Comment:** Some aspects of the manuscript puzzle me, however, and I have some concerns.

First, from the Introduction it is unclear what the rationale is for a preclinical PRISMA extension. Please describe for what aspects preclinical systematic reviews are different from clinical systematic reviews, and why these aspects need additional reporting items?

**Response: We agree with Dr. Langendam that further clarity is needed. Some elaboration has now been added to the introduction to address this issue.**

**“There are fundamental differences between preclinical and clinical systematic reviews of interventions. For instance, subjects are laboratory animals instead of humans, and studies employ different procedures compared with clinical studies (e.g. inducing the disease in animals to mimic the human condition, sacrificing animals to evaluate outcomes, etc.). Therefore, species and strain specific effects, along with unique elements of data abstraction may need to be considered. In addition, preclinical systematic reviews are sometimes performed as precursors to attempted clinical translation of a novel therapy. Therefore, in addition to risk of bias, exploring construct validity of included primary studies (i.e. how well the animal models mimic the disease of interest) may provide important insights on the potential for translation.[9] Finally, it is commonplace to include primary studies with multiple experiments, each with many experimental arms; this requires both significant efforts to identify specific data to include, as well as special considerations for how data will be handled for analysis.”**

**Comment:** Second, the authors developed a 46-item reporting checklist, based on the PRISMA 2009 checklist. Please report what the additional items were? How was decided to include these items, what was the methodological process?

**Response: It is important to distinguish the checklist used here from the eventual PRISMA extension that we are in the process of developing; they are separate. The process of performing a cross-sectional study prior to guideline developments mirrors methods used by Moher *et al^2^* when creating the original PRISMA guidelines and follows best practices for guideline development^3^.**

**The creation of the checklist used here for the reporting assessment was a collaborative and iterative process, wherein all co-authors with expertise in preclinical systematic reviews met on multiple occasions to discuss options for inclusion and exclusions, as well as feedback and input on the checklist.**

**This process was previously described in the Methods. Based on Dr. Langendam’s question we have now elaborated this further:**

**“To create the checklist for the reporting assessment, we consulted PRISMA 2009,[10] along with 4 sets of expert guidance in preclinical systematic review. [3, 11-13] All items from each source were included in a omnibus draft checklist, where each item was framed as one or more “binary” items (that could be answered with a clear yes or no rather than having multiple questions or conditions). This list was compared against additional sources: PRISMA 2020 (pre-print) [14] and several previously published assessments of reports of preclinical systematic reviews [4, 5, 15] to generate a list of items. Items from the list that appeared only relevant to clinical systematic reviews were further evaluated by our team. If these items could not be modified for the preclinical context they were removed. After discussion and feedback from experts in the fields of systematic review reporting and preclinical research, a final checklist containing 46 items (51 with included sub-items) was generated. This was a collaborative and iterative process including all co-authors over the course of several virtual meetings. It is important to note that this checklist is not intended to be an extension for PRISMA, but rather a list of key reporting items our team wanted to assess.”**

**Comment:** Overall it is unclear if the 46-item checklist should be seen as the PRISMA extension, or how the presented checklist will be developed further, making the results in the current manuscript preliminary. The protocol suggests a large research project, how does this manuscript fit in the larger project?

**This assessment is a starting point and a building block to the eventual creation of a PRISMA extension. This first assessment is meant to act as a launching pad for the overall PRISMA preclinical extension program. A much broader list of items will be considered through the planned Delphi consensus process (that will include a much larger group than the current co-author group). “It is important to note that the reporting assessment described here is not an extension to PRISMA; instead, the results of his study will be used to inform the development of the aforementioned preclinical extension (Delphi protocol: https://osf.io/4wy3s/).”**

**Comment:** Third, did the authors analyze trends over time in the characteristics, and if so, what where the results? Is there any improvement? Did they analyze the associations between the general characteristics (for example the type of intervention) and reporting?

**Response: We have now added a formal comparison (z-test) of our sample to the previous assessment of reporting by Mueller et al 2014.^1^ These statistics are now included in the Discussion. With regards to the second half of the comment, we did not analyze any association between general characteristics and reporting.**

**“Mueller et al [5] found that 62% of the reviews they identified provided a flow diagram of included studies; we found that 86% did so (Z-test, p<0.0001). Similarly, reporting of funding also improved (78% vs 38%; Z-test, p<0.00001). Fourteen percent of reviews assessed publication bias in the previous reporting assessment, while 25% of reviews reported the results of publication bias in our updated evaluation (Z-test, p=0.01).”**

**Comment:** Fourth, the authors make a too strong conclusion. The authors conclude that although a considerable number of preclinical systematic reviews have been conducted, their quality and rigour is inconsistent. The authors describe only if some quality-related characteristics, for example risk of bias assessment, were reported. They did not assess if the correct risk of bias instrument was used and applied in an appropriate way. In other words, reporting, not methodological quality was assessed.

**Response: We agree with Dr. Langendam that we assessed quality and completeness of reporting, not methodological rigour. The language has been modified in the abstract and introduction to reflect this important distinction.**

**Abstract:**

**“Our results demonstrate that a considerable number of preclinical systematic reviews have been conducted with diverse topics investigated, however their quality of reporting is inconsistent.”**

**Introduction:**

**“They found the quality of reporting in these reviews low, the majority not reporting assessing the risk of bias or heterogeneity of included studies.”**

**The prevalence and state of reporting of preclinical systematic reviews has not been formally evaluated since 2014 (by Mueller et al,).”**

Minor concerns:

**Comment:** Line 330: Many of the included reviews reported that they followed systematic review reporting guidelines: what guidance where they referring to?

**Response: We have now extracted this information and reported our findings in the Results:**

**“Of the 110 reviews, 46 (42%) explicitly mentioned following/using a reporting guideline or provided a completed reporting checklist. Forty-five of which reported using the PRISMA 2009 statement, and one used the Meta-analyses Of Observational Studies in Epidemiology (MOOSE) checklist.”**

**Comment:** The search date is March 2019, please consider an update of the search.

**Response:** **We would respectfully suggest an update may not be warranted since there have been no major initiatives to address preclinical SRs reporting since the search was conducted. Thus, our co-author group believes there is no reason to think that updated results from 2019-2020 would be meaningfully different. Moreover, an update would not be feasible to complete in a short period of time (please note that this project is being conducted by our team without funding) and it is important to complete this particular study as the planned Delphi exercise to develop an extension to PRISMA will require our available resources. Nonetheless, we have now acknowledged this limitation in our Discussion:**

**“A potential limitation is the timeframe of our sample, as we included reviews from 2015 to 2018 inclusively. Though we chose this sample to capture the state of reporting in preclinical systematic reviews after the previous assessment in 2014, we acknowledge that the state of reporting may have changed from 2018; however, it is important to note that no major initiatives to address systematic review reporting have occurred since that time.”**

**Comment:** Non-English systematic reviews are excluded. Please elaborate if this could cause bias, as we know that many non-English systematic reviews are published.

**Response: We certainly agree, and this is now acknowledged as a limitation in the manuscript. Given the goal of the paper was to assess reporting, we felt it would be difficult to include non-English papers since language barriers would hinder interpretation of reporting. Significant resources would be required to adequately assess non-English papers appropriately.**

**Added to our Discussion:**

**“Furthermore, our sample was restricted to systematic reviews published in the English language, which may have caused the omission of relevant data and the introduction of bias.”**

**Comment:** In the manuscript there is no reference to S4, the checklist.

**Response: This has now been inserted in the Methods:**

**“The checklist can be found in the supporting information (S4 Checklist).”**

**Comment:** Line 120: typo, please delete 'was defined'.

**Response: Corrected.**

**Comment:** Regarding the title: it may be me, but I find the term 'epidemiology' a bit odd when the manuscript is about characteristics of systematic reviews.

**Response: This title was specifically chosen to emulate the landmark paper by Moher *et al^2^*, “a Epidemiology and Reporting Characteristics of Systematic Reviews”, published in *PLoS Medicine* in 2007. This publication led to the creation of the original PRISMA guidelines.**

**Reviewer #3: [identifies himself as David Mellor]**

**Comment:** An important improvement to this paper would be to more fully compare this to previous work (notably, Mueller et al 2014) and to evaluate items that have improved, not changed, or perhaps decreased in the past 6 years. You begin to do this in line 340-344 by comparing 3 items between these two time periods, but I think a more comprehensive comparison would be very helpful to users of this information. Ideally, a table that compares the items included by you, by Mueller, and the rates of each item in common would be helpful. I did not make a side by side comparison between the two checklists, but this would have the further benefit of making such a comparison possible and would provide the opportunity for you to explain any differences between the two. This comparison would make later suggestions and next directions more meaningful.

**Response: We agree with Dr. Mellor that some comparison is warranted. As such, we have now included a formal assessment in the Discussion:**

**“Mueller et al [5] found that 62% of the reviews they identified provided a flow diagram of included studies; we found that 86% did so (Z-test, p<0.0001). Similarly, reporting of funding also improved (78% vs 38%; Z-test, p<0.00001). Fourteen percent of reviews assessed publication bias in the previous reporting assessment, while 25% of reviews reported the results of publication bias in our updated evaluation (Z-test, p=0.01).”**

**It is important to note that Mueller et al did not evaluate the exact same set of items we chose to focus on in our assessment. Moreover, unlike our study where we have provided our full checklist that extractors used, it is also not entirely clear how some of their items were operationalized. Thus, outside of some basic items commented on in the Discussion further comparisons may not be fair.**

**Comment:** The authors note four possible reasons for the relative improvement in the past 6+ years (again, I would like to see a better comparison over time to really see if there is much change): increased funding, leadership provided by CAMARADES and SYRCLE, establishment of PROSPERO, and journal endorsement of PROSPERO. I think that those are all reasonable explanations, but is there any more explanation possible? Without a good sense of the magnitude of the trend, it is hard to ascertain how much explanation is really needed or expected, but if the authors provide more comparison and find substantial change, some more explanation of the possible causes would be helpful. Even if that is not possible, the authors could acknowledge that.

**Response: We agree that this is speculative and the wording in the discussion has been modified: “We speculate that the improved reporting of some items may be explained in-part by the increased funding provided to more recent reviews as well as established leadership by groups such as CAMARADES and SYRCLE.”**

**Comment:** I believe the article would be improved with greater assertions on steps that authors, funders, reviewers, and journals should take to further improve the situation. Some possibilities could include journal requirements for systematic reviews to more fully report details (journal mandates are sometimes effective at changing behaviors such as data sharing <https://osf.io/preprints/bitss/39cfb/> but not others such as registration <https://jamanetwork.com/journals/jamainternalmedicine/fullarticle/2727849> or use of reporting checklists <http://journals.plos.org/plosone/article?id=10.1371/journal.pone.0183591> ) recognition for desired behaviors, eg badges <http://journals.plos.org/plosbiology/article?id=10.1371/journal.pbio.1002456> or review of systematic review protocols such as is conducted by Cochrane or Campbell reviews.

**Response: We agree with Dr. Mellor that this is a critical point to consider. We have added some commentary around this in the Discussion:**

**“In addition to the development of reporting guidelines, other initiatives must be considered to improve the state of reporting and quality of preclinical systematics reviews. Journals, funders, and reviewers could contribute to this improvement by advocating and appealing for data sharing, registration of protocols *a priori*, and** **enforcing the use of tools and resources created by SYRCLE, CAMARADES, and similar groups. Moreover, reporting transparency could be improved by moving beyond simple endorsement and instead enforcing the use of a reporting checklist.”**

**Comment:** Data availability: The authors provide their complete search strategy, checklist, and protocol. However, I was not able to easily find any raw, paper-level data. Even if the authors removed the identifying information from the file (which I don't think would be necessary, but if they want to prevent any single paper as being identified as "worst" then perhaps they could do so), such a file could be helpful. For example, correlations between excluded items could reveal meaningful patterns for future work. If the authors did provide it and I am missing it, please forgive me- although I would recommend adding it to the shared drive along with a code book or README file to explain any odd variables.

**Response: We have now added the paper-level reporting assessment data for the sample of 110 reviews. This is provided in the supporting information.**

**“The reporting assessments for the sample of 110 preclinical reviews is located in the supporting information (S5 Table).”**

Minor comments

**Comment:** Line 99: "The prevalence and quality of preclinical systematic reviews has not been formally evaluated since 2014." It is unclear if the authors are referring to Mueller et al 2014 <https://doi.org/10.1371/journal.pone.0116016> or Page et al 2016 <https://doi.org/10.1371/journal.pmed.1002028> (which reviewed the literature up to 2014), or both. My other comments focus a bit on asking the authors to comment on some trends over time, so this comparison will be necessary.

**Response: We agree that it was not clear what article this statement was referring to. We have clarified that we were referring to the article by Mueller et al 2014.**

**“The prevalence and quality state of reporting of preclinical systematic reviews has not been formally evaluated since 2014 (by Mueller et al).”**

**Comment:** Line 106: It would probably be helpful to point directly to the protocol, <https://osf.io/9mzsv/>

**Response: This has now been added (line 108).**

**Comment:** Line 120: Typo: "We defined preclinical was defined as research…."

**Response: This has been corrected.**

**Comment:** Line 123: Please justify the exclusion of in vitro and ex vivo studies (presumably this is to make it comparable with  Mueller et al 2014, but please state.

**Response: The purpose of our research program is to develop guidelines for the reporting of *in vivo* systematic reviews of interventions, therefore we excluded *in vitro* and *ex vivo* reviews, as they are accompanied by their own unique set of considerations. This paper represents the first step in the creation of these *in vivo* guidelines. We have added the following to our methods:**

**“We only considered *in vivo* animal research, as we intend to use the findings of this work to inform guidelines that are specifically for reporting preclinical systematic reviews of *in vivo* experiments.”**

**However, we recognize the importance of reporting guidelines for systematic reviews of *in vitro* and *ex vivo* studies. In fact, there has already been interest amongst our investigator group in the creation of such guidance and this is an area we plan to explore following the completion of the current guidelines.**

**Comment:** Line 162: "We next evaluated the quality of reporting in a random sample of 25% included systematic reviews. This sample size was chosen based on available resources." It would be helpful for other researchers to provide information on time required to score the 110 articles (either here or around line 246). Not providing this information would be acceptable (perhaps time cannot be reasonably estimated if it was not tracked during the screening), as it is a bit beyond the scope of normal reporting expectations, but would nonetheless be a useful piece of information for others.

**Response: We thank Dr. Mellor for this suggestion. We did not track completion time during the actual data extraction process. Therefore, we feel that we are not able to provide a truly accurate determination of the time required to extract data from one study.**

**Comment:** Line 225, Figure 3: It's unclear why this information is provided in a pyramid shape. The shape suggests more meaning than I think is intended by the authors. Of course, rat is the most common animal, thus at the top of the pyramid, but is not necessarily in a different class or tier than mouse, with nearly as many animals. I think the visual of the animal outlines is a nice way to summarize the information but rearranging into a circle or square would remove any suggestion that the levels are meaningful (beyond simple frequency).

**Response: Based on positive feedback from our co-author group, we’ve elected to keep this in the paper. However, Dr. Mellor raises an important point, i.e. the position within the pyramid reflects only the frequency. This has been clarified in our figure legend:**

**“The structure of the pyramid and positioning of each species / class / family reflects their frequency and does not reflect hierarchy.”**

**Comment:** Line 261: "Twenty-two reviews (20%) reported a protocol had been developed a priori…"

**Response: Disappointing indeed. ☹**

1	Mueller, K. F. *et al.* Dissemination bias in systematic reviews of animal research: a systematic review. *PLoS One* **9**, e116016, doi:10.1371/journal.pone.0116016 (2014).

2	Moher, D., Tetzlaff, J., Tricco, A. C., Sampson, M. & Altman, D. G. Epidemiology and Reporting Characteristics of Systematic Reviews. *PLOS Medicine* **4**, e78, doi:10.1371/journal.pmed.0040078 (2007).

3	Moher, D., Schulz, K. F., Simera, I. & Altman, D. G. Guidance for developers of health research reporting guidelines. *PLoS Med* **7**, e1000217, doi:10.1371/journal.pmed.1000217 (2010).

4	Pound, P. & Ritskes-Hoitinga, M. Is it possible to overcome issues of external validity in preclinical animal research? Why most animal models are bound to fail. *J Transl Med* **16**, 304, doi:10.1186/s12967-018-1678-1 (2018).

5	Drude, N. I., Martinez Gamboa, L., Danziger, M., Dirnagl, U. & Toelch, U. Improving preclinical studies through replications. *Elife* **10**, doi:10.7554/eLife.62101 (2021).
